# Prospective Study on Dynamic Postural Stability in Youth Competitive Alpine Skiers: Test-Retest Reliability and Reference Values as a Function of Sex, Age and Biological Maturation

**DOI:** 10.3389/fphys.2022.804165

**Published:** 2022-04-11

**Authors:** Kirsten Kiers, Lynn Ellenberger, Julia Jermann, Felix Oberle, Walter O. Frey, Jörg Spörri

**Affiliations:** ^1^ Sports Medical Research Group, Department of Orthopedics, Balgrist University Hospital, University of Zurich, Zurich, Switzerland; ^2^ University Centre for Prevention and Sports Medicine, Department of Orthopedics, Balgrist University Hospital, University of Zurich, Zurich, Switzerland

**Keywords:** athletes, physical fitness, postural balance, athletic performance, injury prevention, alpine skiing

## Abstract

This study aimed 1) to assess the test-retest reliability of dynamic postural stability index (DPSI) assessments using a ski-specific jump protocol that consists of single-leg landings on a three-dimensional force plate after forward-performed double-leg drop jumps from a box over a hurdle (DJSLLs), 2) to provide reference values for female and male youth competitive alpine skiers; 3) to explore their changes in DPSI over 3 years during adolescence; and 4) to investigate potential associations of DPSI with age and biological maturation. Using three-dimensional force plates, 16 healthy subjects were tested on the same day (test-retest reliability experiment; five test-retest assessments of right leg landings), and 76 youth skiers aged 13–15 years were tested 3 times within 2 years (main experiment; average of two trials per leg each time). The test-retest reliability experiment revealed an ICC(3,1) and 95% CI of 0.86 [0.74, 0.94] for absolute DPSI assessment. The within-subject SEM of absolute DPSI was 16.30 N [13.66 N, 20.65 N], and the standardized typical error was moderate (0.39 [0.33, 0.50]). Both absolute and relative DPSI values were comparable between male and female youth competitive alpine skiers. The mean absolute DPSI in year 1 (195.7 ± 40.9 N), year 2 (196.5 ± 38.9 N) and year 3 (211.5 ± 41.3 N) continuously increased (i.e., worsened) (*p* < 0.001). Mean relative, i.e. body weight force normalized, DPSI values significantly decreased, i.e., improved, from year 1 to 2 (0.42 ± 0.01 vs. 0.36 ± 0.004; *p* < 0.001) and year 1 to 3 (0.42 ± 0.01 vs. 0.36 ± 0.01; *p* < 0.001). Absolute DPSI correlated with age and biological maturation, while no such correlations were found for relative DPSI values. Our findings suggest that DPSI is a reliable and sensitive measure of dynamic postural control during DJSLLs and that relative DPSI improves annually in competitive youth skiers when accounting for body weight. Future work should consider biological maturation testing during the growth spurt, and normalizing to body weight force could be a possible solution.

## Introduction

Competitive alpine skiing is a sport with a relatively high risk of injury (Florenes et al., 2009; Bere et al., 2014b; Haaland et al., 2016; Alhammoud et al., 2020; Fröhlich et al., 2020), even at the youth level (Schoeb et al., 2020). The most frequent health problems among youth and elite skiers are knee injuries, with the anterior cruciate ligament (ACL) being most commonly ruptured (Florenes et al., 2012; Raschner et al., 2012; Westin et al., 2012; Hildebrandt and Raschner, 2013; Müller et al., 2017; Schoeb et al., 2020). The prevention of such severe injuries should therefore play a key role in the training of competitive alpine skiers. The mechanisms of ACL injuries in alpine skiing have been studied over the last few years, with three injury mechanisms described: “slip-catch”, “landing back-weighted” and “dynamic snowplow” mechanisms (Bere et al., 2011a; Bere et al., 2013; Jordan et al., 2017; Spörri et al., 2017). These situations mainly occur during turns and jump landings and are in most cases the result of out-of-balance situations (Bere et al., 2011a; Bere et al., 2011b; Bere et al., 2014a).

With respect to the mediolateral direction, a skier gets out-of-balance if the force vector resulting from gravitational force and centrifugal force does not direct through the area of support that is spanned by the two skis and the standing width (Howe, 2001; Reid et al., 2020). To maintain balance in the anteroposterior direction, skiers need to react to disturbances due to rapidly changing ski-snow interactions and air resistance, as well as perturbations caused by uneven snow surfaces and gate contacts while skiing in a certain direction, and to bring the body back into dynamic equilibrium as quickly as possible (LeMaster, 2009; Gadient et al., 2010). In the vertical direction, skiers need to compensate for the accelerations and cushion the impacts resulting from convex terrain transitions (compressions) or landing from jumps (Heinrich et al., 2014; Gilgien et al., 2015).

**FIGURE 1:**
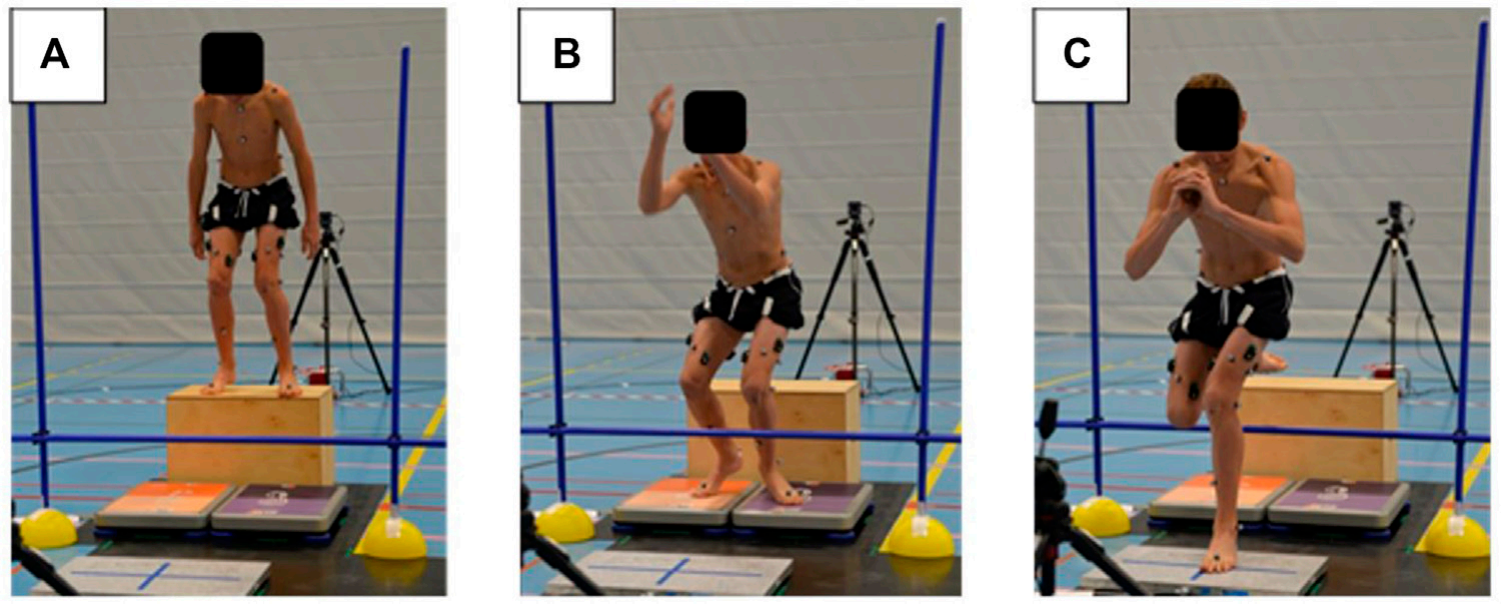
Youth competitive alpine skier performing a DJSLL **(A)** starting on the box **(B)** dropping off the box and landing on both feet and **(C)** jumping over the hurdle with a single leg landing on the force plate in front.

Such skiing-specific balance patterns require excellent neuromuscular control in the lower extremities and trunk to achieve sufficient dynamic postural control under the various challenging and often unpredictable constantly changing conditions of alpine skiing. Youth skiers are especially worthy of investigation, since they are known to have fluctuating neuromuscular control performance during the growth spurt (Quatman-Yates et al., 2012). Because there is no direct equipment connection between the left and right leg as in snowboarding, for instance, the aforementioned skiing-specific dynamic postural control tasks need to be performed unilaterally and independently on each leg. As such, there are certain similarities with the motion task of a single-leg landing on a force plate after a forward-performed double-leg drop jump from a box over a hurdle, hereinafter called drop jump single leg landing (DJSLL), which challenges the dynamic equilibrium in all three directions (Figure 1).

A suitable measure to quantify the balance performance during DJSLL could be found in the Dynamic Postural Stability Index (DPSI), which has already been described several times in previous studies (Goldie et al., 1989; Wikstrom et al., 2005; Hellmers et al., 2017). The DPSI is a measure that determines a person’s ability to regain balance while transitioning from a dynamic to a static state of single-leg stabilization. Over a 3-s period after impact, the fluctuation of ground reaction force (GRF) around the origin during landing is determined, thereby quantifying neuromuscular control in the anteroposterior, mediolateral, and vertical directions (Wikstrom et al., 2005). Previous studies assessed dynamic postural control primarily during basic stance positions, counter movement jumps or single leg landings after two-leg vertical jumps (Goldie et al., 1989; Wikstrom et al., 2005; Hellmers et al., 2017) and reported the latter test to be highly reliable (intraclass correlation coefficient = 0.96) and very precise (SEM = 0.03) (Wikstrom et al., 2005).

Despite the aforementioned research related to DPSI in athletes, to date, no study has analysed DPSI in youth competitive alpine skiers and/or using the slightly modified, forward-performed DJSLL jumping task. Overall, such a task might better reflect the high demands on skiers’ unilateral and leg-independent dynamic postural control mechanisms, in which their centre of mass moves steadily forward while their skis repeatedly and leg-independently change frictional resistance as they glide down the slope (Lind and Sanders, 2004). However, the test-retest reliability of such an alternative test protocol is currently unknown.

Therefore, the aims of the current study were 1) to study the test-retest reliability of DPSI assessments using a ski-specific jump protocol that consists of single-leg landings on a three-dimensional force plate after forward-performed double-leg drop jumps from a box over a hurdle (DJSLLs); 2) to provide reference values for female and male youth competitive alpine skiers; 3) to explore their changes in DPSI over 3 years during adolescence; and 4) to investigate potential associations of DPSI with age and biological maturation.

## Materials and Methods

### Study Design, Setting and Participants

#### Test-Retest Reliability Experiment

To verify the test-retest reliability of DPSI assessments during DJSLL, a cross-sectional study was set up with 16 young, healthy adults (9 females and seven males) who consecutively completed a DJSLL test 5 times on the same day. All instructions and the underlying measurement setup were identical to the “main experiment”. The only exception was the number of repetitions and leg-related unilaterality of the data underlying the reliability calculations (see below). Recruitment of subjects took place via public tender ahead of the experiment. The inclusion criteria were 18–40 years of age and being physically active (i.e., at least 30 min of moderate activity per day or one-time intense physical activity per week). Exclusion criteria were BMI greater than 45 or inability to perform DJSLLs without restrictions due to pain or injury. Participants’ detailed characteristics are described in the corresponding paragraph of the results section.

#### Main Experiment

Out of the potential pool of approximately 220 youth competitive alpine skiers of the U16 category in Switzerland, 76 skiers annually performed the DPSI tests in three consecutive years. In contrast to the test-retest reliability experiment, a total of four trials (2 left leg landings and two right leg landings) were performed and used for further analysis. Skiers were eligible for study participation if they were members of a regional performance centre (RLZ) certified by Swiss-Ski. In Switzerland, children typically start skiing at age 3, participate in their first fun competitions at age 6, and enter a semiprofessional youth development programme with sport-specific training plans at age 12. Each year, tests were conducted before the start of the youth alpine competition season in November. None of the participants were excluded from the study due to predefined exclusion criteria (i.e., health problems that limit current load tolerance, being in the return-to-sport process, or systemic diseases such as inflammatory arthritis or diabetes mellitus). The detailed characteristics of the participants are further described in Table 1 in the results section.

**TABLE 1:** Baseline characteristics of the youth competitive alpine skiers at the beginning of the study.

	Overall (n = 76)	Females (n = 30)	Males (n = 46)
Age [y]	13.7 ± 0.6 (12.5–14.9)	13.6 ± 0.7 (12.5–14.9)	13.8 ± 0.5 (12.9–14.8)
Body height [cm]	161.0 ± 8.0 (143.0–185.0)	160.3 ± 6.4 (143.0–171.5)	161.4 ± 8.9 (146.0–185.0)
Body weight [kg]	48.5 ± 10.1 (30.0–81.0)	46.8 ± 7.6 (35.0–66.0)	49.7 ± 11.4 (30.0–81.0)
BMI [kg/m^2^]	18.5 ± 2.4 (13.0–24.7)	18.1 ± 1.9 (14.4–23.4)	18.8 ± 2.6 (13.0–24.7)
Maturity offset [y]	0.2 ± 1.2 (-2.0–2.8)	1.3 ± 0.7 (0.1–2.8)	-0.5 ± 0.8 (-2.0–1.6)
APHV [y]	13.5 ± 1.1 (11.3–15.4)	12.4 ± 0.5 (11.3–13.4)	14.3 ± 0.6 (12.8–15.4)

Data are expressed as the mean ± SD (minimum-maximum). BMI, body mass index; APH, age at peak height velocity.

#### Ethical Approval

Participation was voluntary, and all participants signed an informed consent form before taking part in this study. For participants under 14 years of age, legal guardians signed the forms. The current study was approved by an institutional review board and the local ethics committee (KEK-ZH-NR: 2017-01395). All procedures were in accordance with the Declaration of Helsinki and national laws.

### Data Collection and Evaluation

#### Baseline Data and Determination of Biological Maturity

All participants of the test-retest reliability experiment underwent a baseline assessment of age, body mass (0.1 kg increments, Seca, Hamburg, Germany), and body height (0.5 cm, determined by measuring tape). In the youth skiers of the main experiment, in addition to the abovementioned baseline measures, sitting height (0.5 cm increments, determined with a tape measure) was measured during each of the three test sessions. Maturity offset was obtained using the noninvasive, anthropometric method proposed by Mirwald et al. (2002) that predicts the age at peak height velocity (APHV) and which had been validated in athletes, as well as in youth competitive alpine skiers previously (Malina et al., 2007; Müller et al., 2015). To calculate maturity offset, sex-specific formulas were used, building upon the following input data: chronological age, body weight, body height, sitting height, and leg length (body height–sitting height). Maturity offset thereby represents a point in time before or after the age at peak height velocity (APHV).

#### Dynamic Postural Stability Index

Biomechanical data were obtained using a three-dimensional (3D) force plate (Kistler Group, Winterthur, Switzerland) to track ground reaction forces during landing after the DJSLL. The 3D force plate data were collected at a sampling rate of 2000 Hz during the landing phase of 5 seconds. All participants underwent a series of trials, all within one test session on the same day. Participants were instructed to start on a 32 cm high box in an upright position (Figure 1A) and drop themselves from the box. The drop was followed by a double-legged landing (Figure 1B) followed by an immediate forward jump over a 37 cm high hurdle with a one-leg landing in the middle of the 3D force plate (Figure 1C). Participants were allowed to move their hands freely in the jump phase. After the hurdle jump, participants should maintain a rather upright position with their hands together in front of the body and stabilize for 5 seconds. Only the first 3 seconds were used for further analysis, as it was found to be the most meaningful period to assess DPSI (Wikstrom et al., 2005). The trial was invalid if participants 1) initiated the drop from the box by actively jumping off, 2) the force plates were not correctly hit during landing, 3) hesitated upon landing before the hurdle jump or 4) stabilized for less than 5 s after the second landing, lost balance or made a second hop. If the jump was invalid, the trial was repeated until a total of two valid trials for each leg were recorded. Between the trials, there was a recovery time of at least 10–15 s.

Raw data from all analogue channels of the force plate were transferred to MATLAB (MATLAB R2016b, The MathWorks, Inc.) to calculate the DPSI values with customized MATLAB scripts. The ground reaction force data were filtered using a second order Butterworth low pass filter with a cut-off frequency of 200 Hz. All trial data were reduced to a 3 s interval of the landing phase after the hurdle jump, beginning at the time of initial ground contact, which was defined as the point in time where the vertical GRF crossed a threshold of 25 N. Absolute DPSI was then calculated over the corresponding interval as a composite of force in all three directions, which are mean square deviations assessing fluctuations around a zero point, with the formula according to Hellmers et al. (2017):

$$Absolute\;DPSI=\sqrt{\frac{\sum {\left(0-F_{x}\right)}^{2}+\sum {\left(0-F_{y}\right)}^{2}+\sum {\left(F_{BW}-F_{z}\right)}^{2}}{n}}$$

*n* represents the number of data points (i.e., 2000 Hz × 3 s = 6000 frames in our study), *F*_*BW*_ is the body weight force, and *F*_*x*_, *F*_*y*_, *F*_*z*_ are the forces in the anteroposterior (x), mediolateral (y) and vertical (z) directions. The relative DPSI was determined by dividing the absolute DPSI by body weight force.

### Statistical Analysis

#### Test-Retest Reliability Experiment

The assessment of the absolute DPSI test-retest reliability was based on five trials consecutively performed on the same day, and only data from the right leg were used. The Shapiro–Wilk test, graphical techniques (i.e., histograms and quantile-quantile plots) and shape parameters (i.e., skewness and kurtosis coefficients) were used to assess the normal distribution of the data. The dependent absolute DPSI differences between the five test repetitions of the 16 young, healthy adults were tested for significance using a repeated-measures ANOVA (*p* < 0.05). These analysis steps were performed in IBM SPSS software (Version 26). For the calculation of the within-subject raw standard error of measurement (SEM, also called “typical error”), within-subject standardized SEM (namely, “standardized typical error”), and the interclass correlation coefficient ICC(3,1) of absolute DPSI assessments during DJSLL, the consecutive pairwise spreadsheet of Hopkins (2015) and the values of five repeated DPSI tests were used. This spreadsheet allows an appropriate test-retest reliability assessment of performance tests where habituation is an issue. SEM was calculated as the degrees of freedom-weighted average of the typical errors (i.e., the SD of the score differences between two adjacent consecutive trials divided by √2) across the five repeated trials assessed (Hopkins, 2015). With respect to the ICC(3,1) calculation, “3” refers to the type of ICC in which the subjects are a random effect and the trials are a fixed effect, while “1” refers to the reliability of single repeated measurements (not the mean of several measurements) (Hopkins, 2015). For the classification of the standardized typical errors, values were doubled before interpreting their magnitude in relation to the common thresholds of 0.2, 0.6, 1.2, 2.0, and 4.0 for the labels *small*, *moderate*, *large*, *very large*, and *extremely large*, as proposed by Smith and Hopkins (2011). The interpretation of the ICC values was based on the classification of Koo and Li (2016) (<0.5, *poor* test-retest reliability; 0.5-0.75, *moderate* test-retest reliability; 0.75–0.9 *good* test-retest reliability; and >0.9 *excellent* test-retest reliability).

#### Main Experiment

The absolute and relative DPSI values of a youth competitive alpine skier, on which further analysis of the main experiment was based, were derived by averaging his/her four performed trials (i.e., two trials with landing on the left leg and two trials with landing on the right leg) into representative mean values. Normal distribution of the data was checked using the Shapiro–Wilk test, graphical techniques (i.e., histograms and quantile-quantile plots) and shape parameters (i.e., skewness and kurtosis coefficients). All accompanying baseline and biological maturity data were evaluated with respect to sex (female vs. male) and time (year 1 vs. year 2 vs. year 3) differences by the use of a multivariate analysis of variance (MANOVA) for repeated measures at *p* < 0.05, with Bonferroni correction for pairwise comparisons. Sex (female vs. male) and time (year 1 vs. year 2 vs. year 3) differences in absolute and relative DPSI values were analysed by two-way repeated-measures analyses of variance (ANOVAs) at *p* < 0.05; again, with Bonferroni correction. Pairwise comparisons were additionally illustrated by mean and 95% confidence interval (CI) plots. Correlations of absolute and relative (i.e., body weight force normalized) DPSI with age and biological maturation were tested using Pearson’s correlation coefficients (r) and coefficients of determination (*R* ^2^). The level of significance was set at *p* < 0.05. IBM SPSS software (Version 26) was used for statistical analysis.

## Results

### Test-Retest Reliability

On a group level, the repeated-measures ANOVA revealed no significant differences in absolute DPSI between the five repetitions at *p* < 0.05 (235.6 ± 40.2 N, 237.5 ± 41.9 N, 232.2 ± 38.3 N, 235.8 ± 49.4 N, 227.8 ± 36.1 N). The test-retest reliability of the absolute DPSI assessment during DJSLL was found to be *good* [ICC(3,1) was 0.86 with a 95% CI of (0.74, 0.94)]. The within-subject SEM was 16.30 N [13.66 N, 20.65 N], and the standardized typical error was found to be *moderate* [0.39 (0.33, 0.50)].

### Baseline Data and Biological Maturity

The baseline characteristics of the young, healthy adults participating in the test-retest reliability experiment were as follows: age 28.9 ± 4.7 years, body height 171.7 ± 8.4 cm, body weight 68.0 ± 10.9 kg, and BMI 22.9 ± 2.0 kg/m^2^. Baseline characteristics for the skiers that participated in the main experiment can be found in Table 1. A repeated-measures ANOVA showed significant differences on a multivariate level between male and female skiers in the main experiment (*p* < 0.001; partial eta^2^ = 0.962) and between the years assessed (*p* < 0.001; partial eta^2^ = 1.000), and there was an interaction effect for year * sex (*p* < 0.001, partial eta^2^ = 0.485). On a univariate level, body height (*p* = 0.016; partial eta^2^ = 0.077), maturity offset (*p* < 0.001; partial eta^2^ = 0.517) and APHV (*p* < 0.001; partial eta^2^ = 0.693) were significantly different between males and females. Over the years, age (*p* < 0.001; partial eta^2^ = 1.000), body height (*p* < 0.001; partial eta^2^ = 0.748), body weight (*p* < 0.001; partial eta^2^ = 0.851), BMI (*p* < 0.001; partial eta^2^ = 0.718), maturity offset (*p* < 0.001; partial eta^2^ = 0.922) and APHV (*p* < 0.001; partial eta^2^ = 0.266) were significantly different on a univariate level.

### Absolute DPSI

**FIGURE 2:**
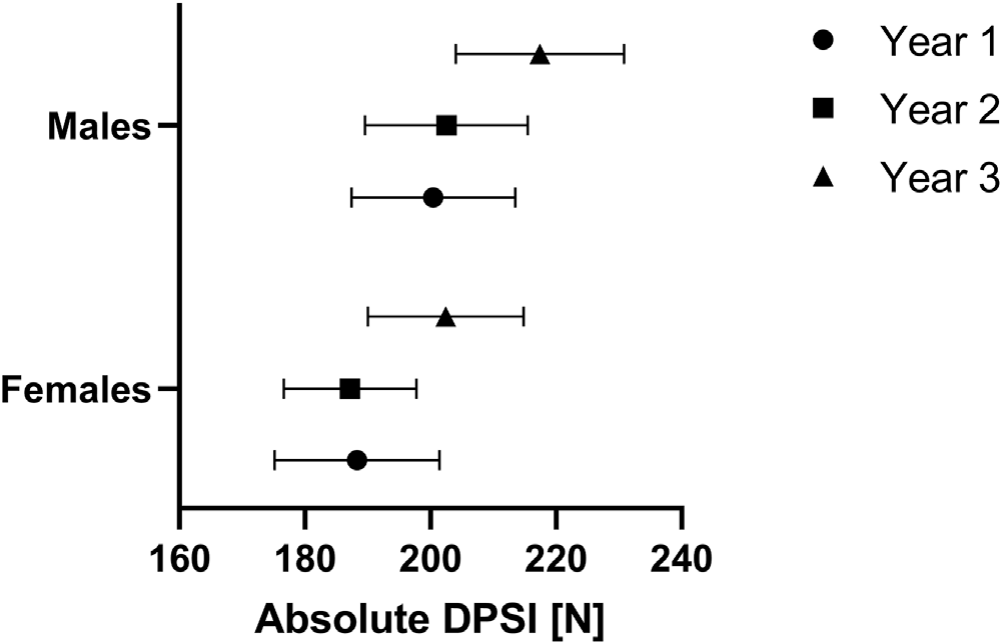
Absolute dynamic postural stability index (DPSI) of youth competitive alpine skiers over 3 years separated by sex. Data are expressed as the mean ±95% CI and represent the sex-specific reference values for the U14, U15 and U16 categories [year 1: females 188.3 N (175.2 N, 201.5 N), males 200.5 N (187.4 N, 213.5 N); year 2: females 187.3 N (176.7 N, 197.8 N), males 202.6 N (189.6 N, 215.6 N); and year 3: females 202.4 N (190.0 N, 214.8 N), males 217.4 N (204.0 N, 230.8 N)].

A repeated-measures ANOVA (sphericity not assumed: Mauchly W (2) = 0.921, *p* = 0.049) showed that absolute DPSI was significantly different over the years (*p* < 0.001, partial eta^2^ = 0.202). Bonferroni corrected pairwise comparisons showed that absolute DPSI was significantly smaller in year 2 (196.5 ± 38.9 N) compared to year 3 (211.5 ± 41.3 N) and in year 1 (195.7 ± 40.9 N) compared to year 3 (211.5 ± 41.3 N), both at *p* < 0.001 (Figure 2). In contrast, absolute DPSI was not significantly different between years 1 and 2 (*p* = 1.000). No significant differences in absolute DPSI values between males and females were observed (*p* = 0.112, partial eta^2^ = 0.034).

### Relative DPSI

**FIGURE 3:**
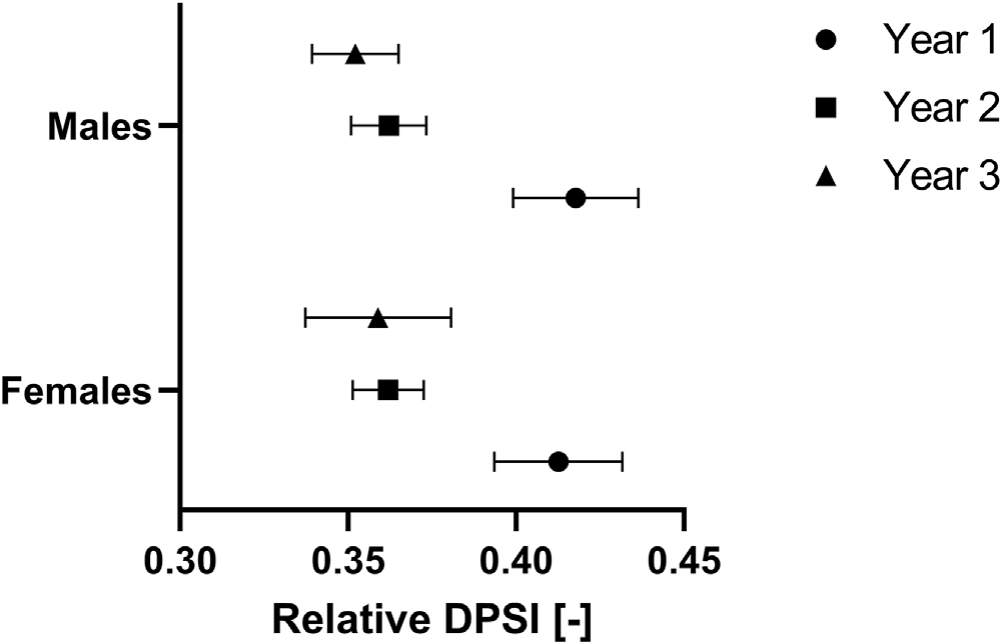
Relative dynamic postural stability index (DPSI) of youth competitive alpine skiers over 3 years separated by sex. Data are expressed as the mean ±95% CI and represent the sex-specific reference values for the U14, U15 and U16 categories [year 1: females 0.41 (0.39, 0.43), males 0.42 (0.40, 0.44); year 2: females 0.36 (0.35, 0.37), males 0.36 (0.35, 0.37); and year 3: females 0.36 (0.34, 0.38), males 0.35 (0.34, 0.36)].

A repeated-measures ANOVA (sphericity not assumed: Mauchly-W (2) = 0.867, *p* = 0.005) shows that the relative DPSI is significantly different over the years (*p* < 0.001, partial eta^2^ = 0.451). Bonferroni corrected pairwise comparisons showed that the relative DPSI was significantly lower in year 2 (0.36 ± 0.03) than in year 1 (0.42 ± 0.06) and in year 3 (0.35 ± 0.05) than in year 1 (0.42 ± 0.06), both at *p* < 0.001 (Figure 3). In contrast, the relative DPSI was not significantly different between years two and 3 (*p* = 0.183). There were no significant differences in the relative DPSI values between males and females (*p* = 0.949).

### Association Between Absolute/Relative DPSI, Age and Maturity Offset

**FIGURE 4:**
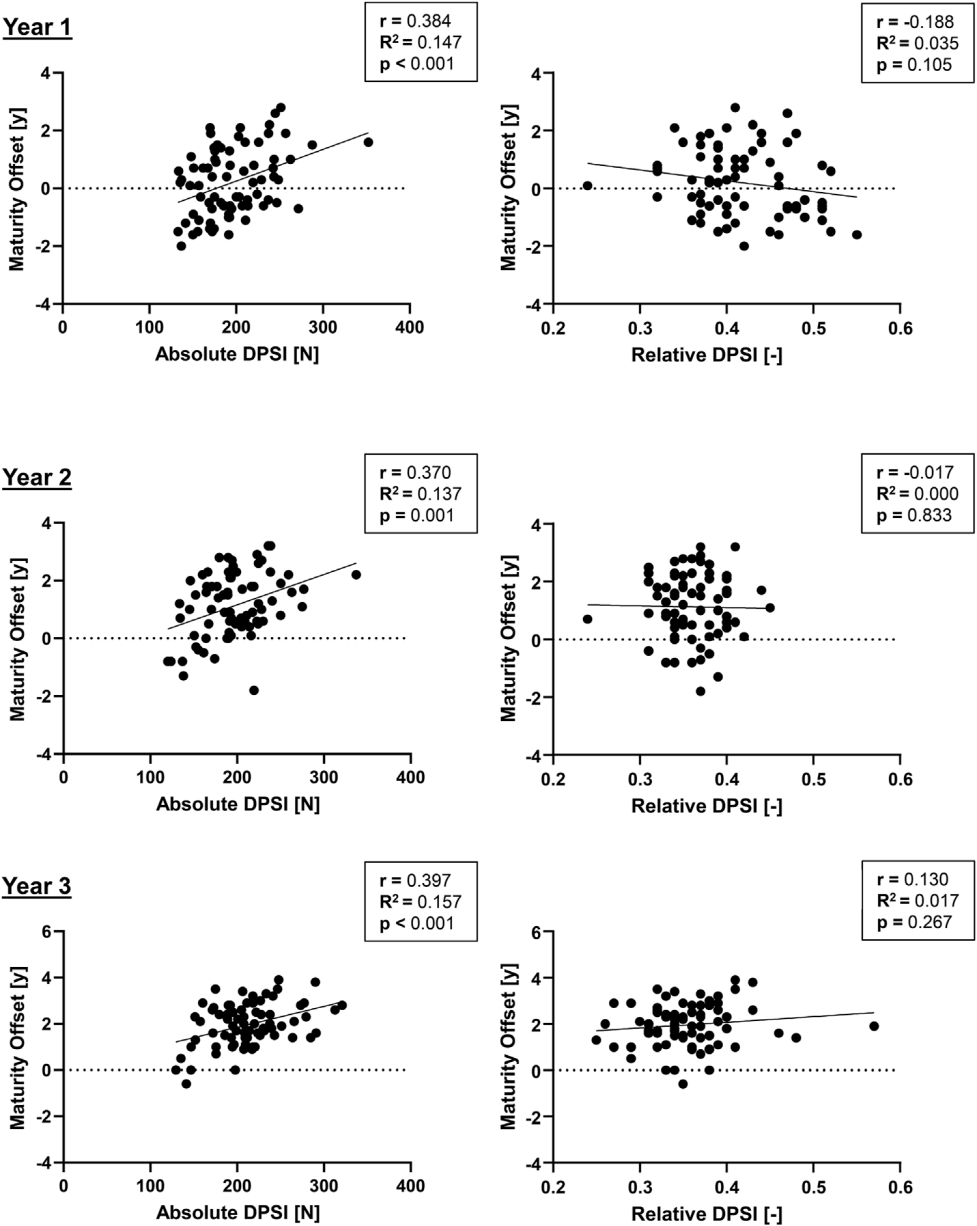
Correlations of absolute and relative dynamic postural stability index (DPSI) with maturity offset in youth competitive alpine skiers.

**FIGURE 5:**
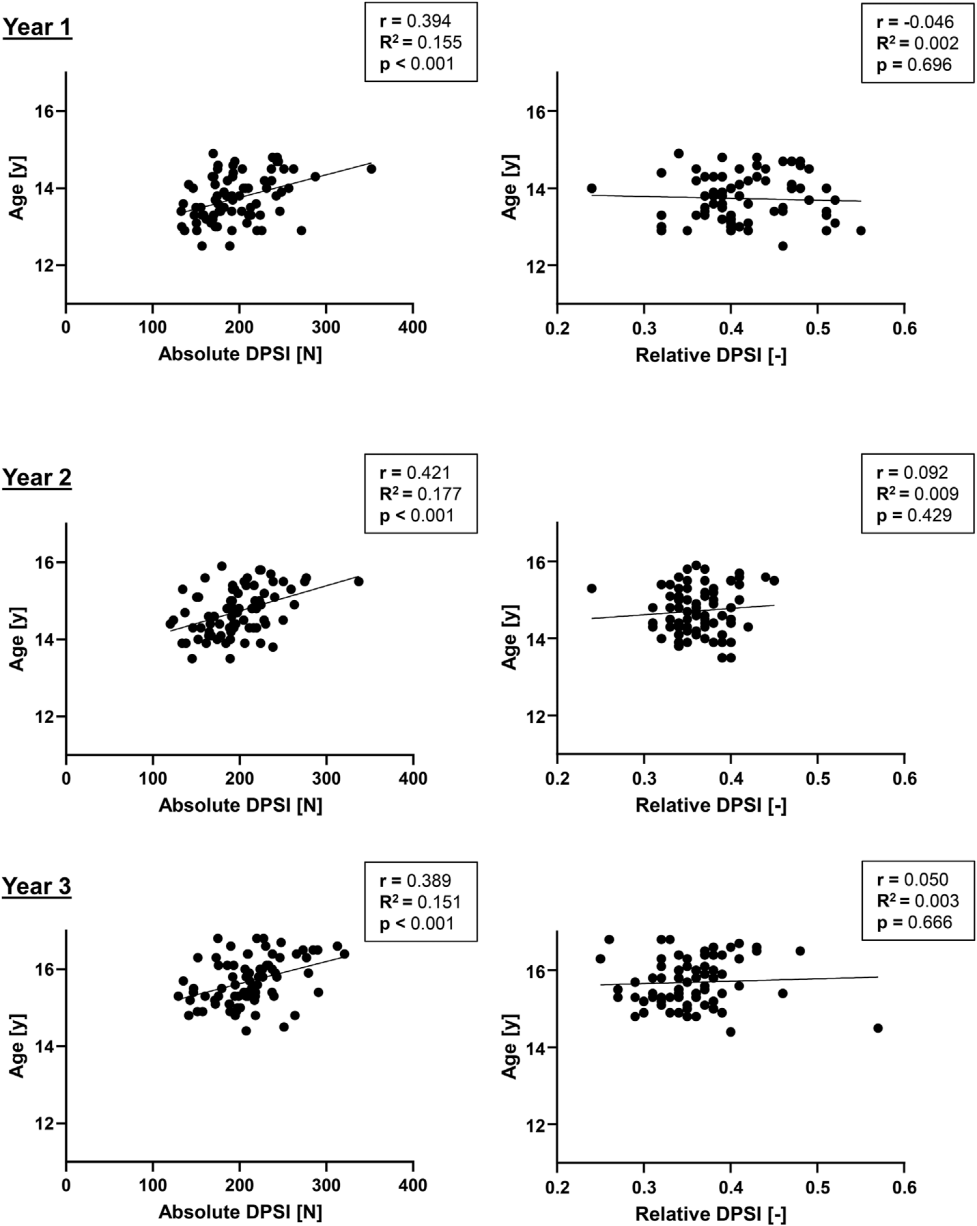
Correlations of absolute and relative dynamic postural stability index (DPSI) with age in youth competitive alpine skiers.

In Figure 4, correlations of absolute/relative DPSI and biological maturity are presented, and correlations of absolute/relative DPSI and age are presented in Figure 5. While for the absolute DPSI values of both sexes over all 3 years, there were significant correlations with biological maturation (*p* < 0.01) and age (*p* < 0.001), such relations were not present for relative, i.e., body weight force normalized, DPSI values.

## Discussion

The purpose of this study was to provide a prospective observation of dynamic postural control ability in 76 youth competitive alpine skiers. The main findings were that 1) the test-retest reliability of absolute DPSI assessments during a jump landing exercise may be considered *good*; 2) both absolute and relative DPSI values were comparable between male and female youth competitive alpine skiers 3) absolute DPSI values increased (i.e., worsened); over 3 years, and relative DPSI values decreased (i.e., improved) when normalized for body weight force; and 4) absolute DPSI correlated with age and biological maturation, while no such correlations were found for relative DPSI values.

### Test-Retest Reliability of Determining DPSI During DJSLL Tasks

The reliability experiment yielded an ICC(3,1) and 95% CI of 0.86 [0.74-0.94] for determining absolute DPSI during DJSLL tasks, which may be considered *good*. In comparison, an earlier study by Wikstrom et al. (2005) reported a slightly better ICC(3,1) of 0.96 [0.91–0.98] for relative DPSI assessments. However, it must also be stressed that the jumping task (i.e., a two-legged jump to a height equal to 50% of the maximum vertical jump and landing on one leg) was probably less challenging and less specific to the demands of alpine skiers than the task included in the current study (including a hurdle jump forward before landing on one leg).

### Sex Differences in Absolute and Relative DPSI Values During DJSLL Tasks

Comparable DPSI values were found for males and females. One may have expected sex differences in DPSI, since males have greater neuromuscular control in general (Hewett et al., 2005; Hewett et al., 2006). During the growth spurt, both males and females gain length and body mass, but sex differences occur around the growth spurt. Puberty hormones affect males and females differently along with the important physical difference that muscle mass and strength increase more prominently in males than in females (Philippaerts et al., 2006). Given this difference in muscle mass, neuromuscular control may differ between males and females, with greater neuromuscular control in males. At the start of this study, female skiers had already passed the growth spurt (1.2 ± 0.7), in contrast to male skiers (-0.5 ± 0.8). The DPSI differences between males and females might therefore be compensated by biological maturity with a lagging neuromuscular control in the male skiers.

### Absolute and Relative DPSI Values Over the three Consecutive Years Around the Growth Spurt

For the absolute DPSI values, a significant increase was observed between years 1–3 and 2–3. When normalizing for body weight force, a significant decrease in relative DPSI values between years 1–2 and 1–3 was revealed. Since lower DPSI values represent good dynamic postural stability per definition, dynamic postural stability seems to worsen over the three consecutive years around the growth spurt when looking just at the absolute values. In contrast, when relative DPSI values are considered, an improvement in dynamic postural stability is observed, with greater improvement between years 1 and 2 than between years 2 and 3. Thus, the increase in body weight between years one to two probably exceeds the decrease in relative DPSI (i.e., the actual improvement in the ability of dynamic postural control), ultimately leading to increased absolute DPSI values (i.e., seemingly worsened dynamic postural control). From a purely theoretical standpoint, this is entirely plausible, given that the absolute DPSI depends on the fluctuations of the measured forces in the *x*, *y* and *z* directions while landing with reference to the values of the same person standing quietly on the force plate, and thereby increases when gaining weight (remember: force (F) is equal to the mass (m) of an object times its acceleration 1) according to the following formula: F = m×a). In addition, it is well known that neuromuscular control changes during the growth spurt, since peak height velocity is the time that the body allows longitudinal growth by the weakening of ligaments, muscles and bones resulting in poor neuromuscular control (Backous et al., 1988; Watkins and Peabody, 1996). Thereby, the legs reach their maximum growth rate before the age at peak height velocity, and in contrast, the trunk reaches its maximum growth rate typically 1 year after the age at peak height velocity (Malina et al., 2004; Philippaerts et al., 2006).

### Correlation of DPSI With Age and Maturity Offset

For the absolute DPSI values of all 3 years, there were significant correlations with age and biological maturation, with the strongest correlation in year two for age (r = 0.421) and in year three for maturity offset (r = 0.397). The corresponding *R* ^2^ values were relatively low, with values up to 0.16 (year 3) for maturity offset and 0.18 for age (year 2), explaining only 16 and 18% of the variability in DPSI. However, when normalized for body weight force, relations between relative DPSI, age and maturity offset were no longer present for all 3 years, suggesting that DPSI is strongly dependent on body weight, which increases during puberty. Regular testing of athletes such as youth competitive alpine skiers is an important part of sports. When testing physical fitness in youth skiers around the growth spurt, performance is dependent on the status of biological maturation and thereby body weight, which makes it challenging to interpret the results with only chronological age as a dependent factor between individuals. In this study, the DPSI values increased but decreased when normalized for body weight force. This further stresses the importance of body weight normalization of physical fitness tests in youth skiers around the growth spurt.

### Study Limitations

First, the methodological approach used in this study to evaluate postural stability in a controlled laboratory environment is only a very rough imitation of the real ski situation, but with significant advantages in terms of standardization of test conditions.

Second, by definition, for the single-leg landings, only trials in which the foot was in continuous contact with the force plate for at least 3 s were valid. However, when the field tests were conducted in the first year, not enough priority was given to on-site monitoring of this aspect, so the number of subjects with three valid annual test scores was significantly limited, which may have led to some selection bias, with subjects with very poor dynamic postural stability abilities being more likely to have been excluded.

Third, since the underlying cohorts of the reliability experiment and the main experiment differ in age and sporting level, direct transferability of the reliability experiment results to the main experiment is limited and should be undertaken with caution. However, the young, healthy adults’ cohort of the reliability experiment may serve as a representative sample for many application contexts of future studies, and at least the DPSI average and standard deviation magnitudes in year three of the main experiment (average absolute DPSI values: 211.5 ± 41.3 N) are comparable to those of the young, healthy adults in the reliability experiment (average absolute DPSI values: 233.8 ± 41.4 N). In year three of the main experiment, the skiers were approaching the age of 16 years, and therefore the anthropometry and balance abilities of the competitive skiers (at this time still semiprofessional athletes) might have been comparable to those of the young, healthy adults.

Fourth, the different number of repetitions and leg-related unilaterality versus bilaterally of the data underlying the reliability and main experiment calculations, respectively, may further limit a direct transferability between the two experiments. However, both experiments had a clearly defined purpose, which may justify the different analysis approaches. The reliability experiment aimed to account for habituation effects while avoiding bias due to increasing fatigue. Conducting five test repetitions on both sides on the same day, instead of five test repetitions on one side on the same day, likely would have introduced increasing exhaustion. The main experiment sought to increase the individual representativeness of the test results while minimizing testing efforts, which is crucial when screening large cohorts of athletes such as youth competitive alpine skiers. Accordingly, in the main experiment, a youth skiers’ representative absolute/relative DPSI values were obtained by averaging his/her four performed trials (i.e., two trials per leg) into a representative mean value. Notwithstanding, the one-sided DPSI values (as described in the reliability experiment) may provide further valuable insight and clearer guidelines for potential countermeasures once a global balance deficit has been identified in a particular skier.

Fifth, despite a clear theoretical and conceptual link between DPSI, skiing performance, and the risk of injury, the present study did not investigate such a potential relationship. This study also did not explore DPSI leg asymmetries and the influence of leg dominance on unilateral DPSI values. Since these aspects have not been the focus of previous studies, they should be the subject of future research.

## Conclusion

The DPSI during DJSLLs may basically be considered a reliable and sensitive measure for assessing dynamic postural control. However, test-retest reliability may differ between specific cohorts, and biological maturation should be considered a potential confounder when interpreting absolute DPSI test values of youth skiers during the growth spurt. This is also apparent, among other things, from the fact that in the present study, an increase in dynamic postural control ability over the years was observed for body weight force normalized, relative DPSI values, whereas a deterioration occurred for absolute DPSI values.

## Data Availability

The datasets presented in this article are not readily available because their access is restricted to protect the interests of the project partner Swiss-Ski and their athletes. Requests to access the datasets should be directed to joerg.spoerri@balgrist.ch.

## References

[B1] Alhammoud M. Racinais S. Rousseaux‐Blanchi M. P. Bouscaren N. (2020). Recording Injuries Only during winter Competitive Season Underestimates Injury Incidence in Elite alpine Skiers. Scand. J. Med. Sci. Sports 30, 1177–1187. 10.1111/sms.13648 32141109

[B2] Backous D. D. Friedl K. E. Smith N. J. Parr T. J. Carpine W. D. Jr. (1988). Soccer Injuries and Their Relation to Physical Maturity. Arch. Pediatr. Adolesc. Med. 142, 839–842. 10.1001/archpedi.1988.02150080045019 3394676

[B3] Bere T. Flørenes T. W. Krosshaug T. Haugen P. Svandal I. Nordsletten L. (2014a). A Systematic Video Analysis of 69 Injury Cases in World Cup alpine Skiing. Scand. J. Med. Sci. Sports 24, 667–677. 10.1111/sms.12038 23301907

[B4] Bere T. Flørenes T. W. Krosshaug T. Koga H. Nordsletten L. Irving C. (2011a). Mechanisms of Anterior Cruciate Ligament Injury in World Cup Alpine Skiing. Am. J. Sports Med. 39, 1421–1429. 10.1177/0363546511405147 21515807

[B5] Bere T. Florenes T. W. Krosshaug T. Nordsletten L. Bahr R. (2011b). Events Leading to Anterior Cruciate Ligament Injury in World Cup Alpine Skiing: a Systematic Video Analysis of 20 Cases. Br. J. Sports Med. 45, 1294–1302. 10.1136/bjsports-2011-090517 22067283

[B6] Bere T. Flørenes T. W. Nordsletten L. Bahr R. (2014b). Sex Differences in the Risk of Injury in World Cup alpine Skiers: a 6-year Cohort Study. Br. J. Sports Med. 48, 36–40. 10.1136/bjsports-2013-092206 23673520

[B7] Bere T. Mok K.-M. Koga H. Krosshaug T. Nordsletten L. Bahr R. (2013). Kinematics of Anterior Cruciate Ligament Ruptures in World Cup Alpine Skiing. Am. J. Sports Med. 41, 1067–1073. 10.1177/0363546513479341 23449837

[B8] Flørenes T. W. Nordsletten L. Heir S. Bahr R. (2012). Injuries Among World Cup Ski and Snowboard Athletes. Scand. J. Med. Sci. Sports 22, 58–66. 10.1111/j.1600-0838.2010.01147.x 20561277

[B9] Florenes T. W. Bere T. Nordsletten L. Heir S. Bahr R. (2009). Injuries Among Male and Female World Cup alpine Skiers. Br. J. Sports Med. 43, 973–978. 10.1136/bjsm.2009.068759 19945979

[B10] Fröhlich S. Helbling M. Fucentese S. F. Karlen W. Frey W. O. Spörri J. (2020). Injury Risks Among Elite Competitive alpine Skiers Are Underestimated if Not Registered Prospectively, over the Entire Season and Regardless of whether Requiring Medical Attention. Knee Surg. Sports Traumatol. Arthrosc. 29, 1635–1643. 10.1007/s00167-020-06110-5 32556431

[B11] Gadient V. Dannenberger D. Homberger M. Kindschi A. Läuppi P. Malär C. (2010). *Schneesport Schweiz, Band 2: Ski.* Belp. Belp, Switzerland: SWISS SNOWSPORTS Association SSSA.

[B12] Gilgien M. Crivelli P. Spörri J. Kröll J. Müller E. (2015). Characterization of Course and Terrain and Their Effect on Skier Speed in World Cup alpine Ski Racing. PLoS One 10, e0118119. 10.1371/journal.pone.0118119 25760039 PMC4356573

[B13] Goldie P. A. Bach T. M. Evans O. M. (1989). Force Platform Measures for Evaluating Postural Control: Reliability and Validity. Arch. Phys. Med. Rehabil. 70, 510–517. 2742465

[B14] Haaland B. Steenstrup S. E. Bere T. Bahr R. Nordsletten L. (2016). Injury Rate and Injury Patterns in FIS World Cup Alpine Skiing (2006-2015): Have the New Ski Regulations Made an Impact? Br. J. Sports Med. 50, 32–36. 10.1136/bjsports-2015-095467 26559877

[B15] Heinrich D. Van Den Bogert A. J. Nachbauer W. (2014). Relationship between Jump landing Kinematics and Peak ACL Force during a Jump in Downhill Skiing: a Simulation Study. Scand. J. Med. Sci. Sports 24, e180–e187. 10.1111/sms.12120 24118532

[B16] Hellmers S. Fudickar S. Dasenbrock L. Heinks A. Bauer J. M. Hein A. (2017). “Understanding Jump Landing as an Oscillating System: A Model-Based Approach of Balance and Strength Analyses,” in 10th International Conference on Health Informatics. 10.5220/0006171101590168

[B17] Hewett T. E. Myer G. D. Ford K. R. Heidt R. S. Jr. Colosimo A. J. Mclean S. G. (2005). Biomechanical Measures of Neuromuscular Control and Valgus Loading of the Knee Predict Anterior Cruciate Ligament Injury Risk in Female Athletes: a Prospective Study. Am. J. Sports Med. 33, 492–501. 10.1177/0363546504269591 15722287

[B18] Hewett T. E. Myer G. D. Ford K. R. Slauterbeck J. R. (2006). Preparticipation Physical Examination Using a Box Drop Vertical Jump Test in Young Athletes. Clin. J. Sport Med. 16, 298–304. 10.1097/00042752-200607000-00003 16858212

[B19] Hildebrandt C. Raschner C. (2013). Traumatic and Overuse Injuries Among Elite Adolescent alpine Skiers: A Two-Year Retrospective Analysis. Int. Sportmed J. 14, 245–255.

[B20] Hopkins W. G. (2015). Spreadsheets for Analysis of Validity and Reliability. Sportscience 19, 36–44.

[B21] Howe J. (2001). The New Skiing Mechanics. Waterford: McIntire Publishing.

[B22] Jordan M. Aagaard P. Herzog W. (2017). Anterior Cruciate Ligament Injury/reinjury in alpine Ski Racing: a Narrative Review. Oajsm Vol. 8, 71–83. 10.2147/oajsm.s106699 PMC5386612 28435336

[B23] Koo T. K. Li M. Y. (2016). A Guideline of Selecting and Reporting Intraclass Correlation Coefficients for Reliability Research. J. Chiropractic Med. 15, 155–163. 10.1016/j.jcm.2016.02.012 PMC4913118 27330520

[B24] Lemaster R. (2009). Ultimate Skiing. Leeds, United Kingdom: Human Kinetics.

[B25] Lind D. Sanders S. P. (2004). The Physics of Skiing - Skiing at the Triple Point. New York: Springer-Verlag.

[B26] Malina R. M. Bouchard C. Bar-Or O. (2004). Growth, Maturation, and Physical Activity, Champaign, Illinois: Human Kinetics.

[B27] Malina R. M. Dompier T. P. Powell J. W. Barron M. J. Moore M. T. (2007). Validation of a Noninvasive Maturity Estimate Relative to Skeletal Age in Youth Football Players. Clin. J. Sport Med. 17, 362–368. 10.1097/jsm.0b013e31815400f4 17873548

[B28] Mirwald R. L. G. Baxter-jones A. D. Bailey D. A. Beunen G. P. (2002). An Assessment of Maturity from Anthropometric Measurements. Med. Sci. Sports Exerc. 34, 689–694. 10.1249/00005768-200204000-00020 11932580

[B29] Müller L. Hildebrandt C. Müller E. Oberhoffer R. Raschner C. (2017). Injuries and Illnesses in a Cohort of Elite Youth alpine Ski Racers and the Influence of Biological Maturity and Relative Age: a Two-Season Prospective Study. Open Access J. Sports Med. 8, 113–122. 10.2147/OAJSM.S133811 28546774 PMC5436787

[B30] Müller L. Müller E. Hildebrandt C. Kapelari K. Raschner C. (2015). Die Erhebung des biologischen Entwicklungsstandes für die Talentselektion - welche Methode eignet sich? Sportverletz Sportschaden 29, 56–63. 10.1055/s-0034-1399043 25710395

[B31] Philippaerts R. M. Vaeyens R. Janssens M. Van Renterghem B. Matthys D. Craen R. (2006). The Relationship between Peak Height Velocity and Physical Performance in Youth Soccer Players. J. Sports Sci. 24, 221–230. 10.1080/02640410500189371 16368632

[B32] Quatman-Yates C. C. Quatman C. E. Meszaros A. J. Paterno M. V. Hewett T. E. (2012). A Systematic Review of Sensorimotor Function during Adolescence: a Developmental Stage of Increased Motor Awkwardness? Br. J. Sports Med. 46, 649–655. 10.1136/bjsm.2010.079616 21459874 PMC4157222

[B33] Raschner C. Platzer H.-P. Patterson C. Werner I. Huber R. Hildebrandt C. (2012). The Relationship between ACL Injuries and Physical Fitness in Young Competitive Ski Racers: a 10-year Longitudinal Study. Br. J. Sports Med. 46, 1065–1071. 10.1136/bjsports-2012-091050 22968156

[B34] Reid R. C. Haugen P. Gilgien M. Kipp R. W. Smith G. A. (2020). Alpine Ski Motion Characteristics in Slalom. Front. Sports Act Living 2, 25. 10.3389/fspor.2020.00025 33345019 PMC7739813

[B35] Schoeb T. Peterhans L. Fröhlich S. Frey W. O. Gerber C. Spörri J. (2020). Health Problems in Youth Competitive alpine Skiing: a 12-month Observation of 155 Athletes Around the Growth Spurt. Scand. J. Med. Sci. Sports 30, 1758–1768. 10.1111/sms.13740 32502323

[B36] Smith T. B. Hopkins W. G. (2011). Variability and Predictability of Finals Times of Elite Rowers. Med. Sci. Sports Exerc. 43, 2155–2160. 10.1249/mss.0b013e31821d3f8e 21502896

[B37] Spörri J. Kröll J. Gilgien M. Müller E. (2017). How to Prevent Injuries in Alpine Ski Racing: What Do We Know and where Do We Go from Here? Sports Med. 47, 599–614. 10.1007/s40279-016-0601-2 27480763 PMC5357247

[B38] Watkins J. Peabody P. (1996). Sports Injuries in Children and Adolescents Treated at a Sports Injury Clinic. J. Sports Med. Phys. Fitness 36, 43–48. 8699837

[B39] Westin M. Alricsson M. Werner S. (2012). Injury Profile of Competitive alpine Skiers: a Five-Year Cohort Study. Knee Surg. Sports Traumatol. Arthrosc. 20, 1175–1181. 10.1007/s00167-012-1921-x 22349602

[B40] Wikstrom E. A. Tillman M. D. Smith A. N. Borsa P. A. (2005). A New Force-Plate Technology Measure of Dynamic Postural Stability: the Dynamic Postural Stability index. J. Athl Train. 40, 305–309. 16404452 PMC1323292

